# Retraction Note: MicroRNA-31-5p regulates chemosensitivity by preventing the nuclear location of PARP1 in hepatocellular carcinoma

**DOI:** 10.1186/s13046-019-1158-3

**Published:** 2019-04-02

**Authors:** Ke-ting Que, Yun Zhou, Yu You, Zhen Zhang, Xiao-ping Zhao, Jian-ping Gong, Zuo-jin Liu

**Affiliations:** Department of Hepatobiliary Surgery, the Second Affiliated Hospital of Chongqing Medical University, Chongqing, 400010 China

**Retraction Note: J Exp Clin Cancer Res**

**https://doi.org/10.1186/s13046-018-0930-0**

The authors are retracting this article [1] because it overlaps significantly with a previously published article by Moody et al. [2] without proper citation. All authors agree with this retraction.
